# Loss aversion in EQ-5D-Y-3L: does it explain differences in willingness to trade-off life years in adults and children?

**DOI:** 10.1007/s10198-025-01775-6

**Published:** 2025-04-12

**Authors:** Ava F. H. Hoogenboom, Stefan A. Lipman

**Affiliations:** 1 Erasmus School of Health Policy & Management, Erasmus University Rotterdam, Burgemeester Oudlaan 50, Rotterdam, 3062 PA The Netherlands; 2 Erasmus Centre for Health Economics Rotterdam (EsCHER), Erasmus University Rotterdam, Burgemeester Oudlaan 50, Rotterdam, 3062 PA The Netherlands

**Keywords:** Loss aversion, EQ-5D-Y, Health state valuation, Time trade-off, Quality-adjusted life year, I10

## Abstract

**Introduction:**

Earlier work has shown that adults valuing health for 10-year-old children (i.e., in a child perspective) are more reluctant to trade-off life duration than for themselves, generating higher utilities in composite time trade-off (cTTO). The main motivation of this study is to explore if this reluctance can be explained through loss aversion, i.e., losses of life duration weighing more than gains of the same size.

**Methods:**

100 UK adults completed cTTO tasks for six EQ-5D-Y-3L states and tasks measuring loss aversion. Both sets of tasks were completed from the child perspective and for the respondent themselves, enabling perspective-dependent correction for loss aversion.

**Results:**

A slight majority of participants was explicitly more loss averse for children than for themselves. Health state utilities were higher in the child perspective both before and after correction for loss aversion. Differences between utilities elicited in both perspectives and the variance of cTTO utilities increased considerably after correction.

**Discussion:**

The results suggest that loss aversion does not explain differences in willingness to trade-off life duration between perspectives. Hence, it remains unclear if correction for loss aversion should be recommended when using EQ-5D-Y-3L utilities in practice.

**Supplementary Information:**

The online version contains supplementary material available at 10.1007/s10198-025-01775-6.

## Introduction

Many countries in the world make policy choices in healthcare based on or informed by economic evaluations [1, 2]. In these evaluations, the costs and benefits of healthcare interventions are compared to plausible alternatives. Often, the benefits are expressed in terms of quality-adjusted life years (QALYs), which entails weighting life years gained (or lost) through healthcare interventions with ‘utilities’ [3] that represent the health-related quality of life in which those years are spent. Utilities are normalized such that being dead has a weight of 0 and full health has a weight of 1.

The standard practice in the Netherlands and many other countries is the use of EQ-5D instruments to elicit utilities for health states [4]. These questionnaires are designed for patients to self-report their state of health using five dimensions: mobility, self-care, usual activities, pain/discomfort, and anxiety/depression. Respondents rate their health on these dimensions using multiple (e.g., 5 for EQ-5D-5L) levels of severity ranging from no problems to extreme problems [5]. Utilities can be attached to the EQ-5D health states with the help of previously developed value sets, which collect a countries’ general public’s preferences for EQ-5D health states for themselves [6–8].

An EQ-5D questionnaire was developed to facilitate decision-making regarding paediatric healthcare. This EQ-5D-Y-3L instrument is an adapted questionnaire recommended for use with children aged 8 to 15 [9]. Completing this questionnaire results in a description of the associated health state in numbers. For example, if you rate your health on all dimensions at the lowest level of severity, your health state would be 11111. The EQ-5D-Y-3L has three severity levels, meaning that the health state with the highest severity on all dimensions would be 33333 [10]. Whereas for adult EQ-5D instruments these tasks are completed by adults who value hypothetical health states for themselves, for EQ-5D-Y-3L adults are asked to complete these tasks considering their views of a 10-year-old child.

As such, the perspective used for valuation of EQ-5D-Y-3L differs from that used in adult versions of EQ-5D. The rationale and consequences of this change between what are henceforth referred to as the adult and the child perspective have been a source of ongoing inquiry and discussion [11–14]. Lipman, Reckers-Droog, Karimi, Jakubczyk and Attema [15] identify two channels to which this could lead to changes in individuals’ choices: (i) a respondent is now valuing child health states instead of adult health states and (ii) a respondent is now valuing somebody else’s health state instead of their own. As such, two questions now arise: what happens when people make decisions on behalf of someone else? And, what happens when adults make decisions on behalf of a child?

Some evidence exists suggesting that both channels could affect decision-making. For example, it has already been found that people tend to make riskier choices when a decision concerns another person than when it concerns themselves [16]. In addition, several studies have found that people prioritize healthcare for children over healthcare for adults. Though the reasons why seem to differ across countries and across studies, many researchers have found evidence for preferences for health gains in children over gains in adults [17–21]. When focusing on health state valuation in particular, researchers also expect differences when people make decisions from different perspectives [15, 22, 23]. More specifically, researchers have found differences in TTO valuations between the child and adult perspective. Overall, utilities seem to be higher for the child than for the adult perspective [15, 22–24]. These differences in utilities have consequences for QALY estimations, which in turn have implications for economic evaluations [25].

Several studies have explored why the differences between perspectives exist. Reckers-Droog, Van Exel and Brouwer [26] found that the willingness to pay for quality-of-life gains of the Dutch public is higher for younger patients, suggesting that they view child health differently than adult health. A qualitative study by Åström, Conte, Berg and Burström [11] found people to be reluctant to give up life years when valuing children’s health states. “Child life-years are considered more precious” [12]. Hence it seems that the value of life duration may differ between perspectives, with children’s life duration being more valuable than adult health. Lipman, Zhang, Shah, and Attema [24] studied these differences by exploring differences in discounting (i.e., the rate at which health in the future decreases in value relative to health in the present) between both perspectives. If people discount child health differently than their own health, this might explain some of the differences between the perspectives in TTO tasks. Lipman et al. [24] in fact found that in both perspectives respondents were inclined to negatively discount health (e.g., they value health in the future more than health now). Moreover, Lang, Attema and Lipman [27] show that adult-child differences in TTO utilities disappear when correcting for time preference. Yet, Bleichrodt [28] suggested that in TTO tasks, the value of life duration is not only affected by discounting, but also by a phenomenon called loss aversion. Loss aversion implies that people attach more weight to losses than to gains of the same size, which will yield bias in TTO tasks [28].

Researchers from various fields have studied loss aversion. For instance, it has been found that people are indeed loss averse when it comes to income [29, 30]. Empirical research suggests that loss aversion also affects health state valuation, meaning that people tend to attach more weight to health sacrificed than to potential gains when completing health valuation tasks such as TTO. As a consequence, people are reluctant to sacrifice life duration. When this reluctance is not accounted for, TTO utility weights are biased upwards– meaning that the resulting valuation of health states is higher than it should be [31–34].

Earlier work provides some reasons to expect that loss aversion differs between perspectives and thus that loss aversion may explain the differences between the adult and the child perspective. That is, earlier literature provides support for three distinct observations: (1) loss aversion introduces an upward bias in TTO utilities [ 33], (2) several researchers have found that loss aversion actually decreases when making decisions on behalf of someone else [35–38], and (3) as mentioned before, several qualitative studies suggest that when it comes to children, people tend to be reluctant to give up life years [11, 12], which might lead to inflated utilities. This unwillingness to sacrifice life duration may be caused by loss aversion (or, alternatively, may be modelled by a measure of loss aversion). At the time of writing, no studies on the effect of loss aversion on EQ-5D-Y-3L valuation exist, which is why this study will focus on exploring whether this could (partly) explain the differences in utilities elicited from the adult and child perspective.

The main motivation of this explorative study therefore is to explore if loss aversion can (partly) explain the differences between health state valuations completed from the perspective of an adult and valuations completed from the perspective of a 10-year-old child when using time trade-off. If differences are indeed found that can (partly) be explained by loss aversion, this would have implications for the utilities derived with the EQ-5D-Y-3L and could suggest that corrections of these utilities are needed (as developed in Lipman, Attema & Versteegh [33]). The remainder of this paper is structured as follows: the QALY model and the incorporation of loss aversion are described in the theoretical framework (Sect. 2). Section 3 presents how data was collected and analysed. The analyses and data are shown in Section 4 and the discussion (Sect. 5) describes several conclusions and limitations of this study.

## Theoretical framework

Health state valuation typically assumes that the general QALY model holds [39]. This model expresses the value of health profiles (T, Q):

1 $$V\left( {T,Q} \right) = L\left( T \right)U\left( Q \right)$$

in which (T, Q) represents living in health status Q for T years. L(T) is the utility associated with the life duration and U(Q) is the utility associated with health state Q.

Although TTO tasks can in principle be completed with any duration, in EQ-5D valuation, TTO tasks involve elicitation of the indifference between 10 years in health state Q and living T years in full health. The value for T is elicited through a series of choices, yielding the following indifference (represented by ~):

2 $$\:\left(10,Q\right)\sim\:\left(T,\:FH\right).$$

In QALY models, it is standard practice to define U(FH) = 1, which means we can derive the utility of health state Q using Eq. [3]:

3 $$\:U\left(Q\right)\:=\:L\left(T\right)/L\left(10\right)$$

In practice (i.e., in nearly all applications of TTO), we often assume instead the linear QALY model holds, which implies that L(T) = T, resulting in Eq. [4]:

4 $$U\left( Q \right) = T/10$$

These ‘standard’ TTO questions can only be used for health states that are perceived as better than being dead (BTD states), as for all states worse than dead (WTD) duration T would take a value of 0 (i.e., people prefer not living). If a health state is perceived to be WTD, a lead-time TTO question can be asked to allow for disutility for Q. That is, the task will be focused on eliciting the indifference between living in full health for a period of 10 years followed by a period of 10 years in health state Q (which we denote as $\:(10,FH;10,Q))$, and living T years in full health [24]:

5 $$\:(10,FH;10,Q)\:\sim\:(T,\:FH)$$

Using U(FH) = 1 and L(T) = T, this leads to the following Eq. [33]:

6 $$\:U\left(Q\right)=(T-10)/10\:$$

This combination of standard and lead-time TTO questions for BTD and WTD states is called composite TTO (cTTO). Lipman, Attema and Versteegh [33] present a QALY model based on prospect theory that incorporates loss aversion (*λ*) and show how cTTO can be evaluated within that model. In the current study, only those final evaluations (and the intuition underlying them) are reported for the sake of brevity. Any model based on prospect theory requires the definition of a reference-point, which separates gains from losses. Lipman, Attema and Versteegh [33] assume that in TTO exercises the 10 years in Q serve as the reference-point on which people base their choices, as this is the guaranteed lifetime they compare the alternative (T, FH) with. Life duration sacrificed compared to that reference-point receives an extra weight *λ* to reflect loss aversion. Formally, we use the same approach and normalisation as Lipman, Attema and Versteegh [33], which incorporates loss aversion in the general QALY model evaluation of ‘standard’ TTO indifferences (as in Eq. 2) as follows:

7 $$\:U\left(Q\right)=\frac{L\left(T\right)}{\lambda\:L\left(10\right)+\left(1-\lambda\:\right)L\left(T\right)}$$

in which $\:\lambda\:$ denotes the loss aversion coefficient with $\:\lambda\:$ >1 characterizing loss aversion [33]. Note that L(T) here is normalized such that L(20) = 1 and L(0) = 0, which means that to solve Eq. [7] we need to measure L(T), L(10) and $\:\lambda\:$. Earlier work has already explored how the shape of L(T) affects the difference between TTO utilities elicited with an adult and child perspective [24, 27]. Hence, in this paper, we focus only on the effect of loss aversion, and, as such, will (for simplicity) assume that L(T) is linear. We do still maintain the normalisation Lipman et al. (2022) applied. As such L(T) is scaled such that L(T) = T/20 and L(10) can be calculated using L(10)  = 10/20 [33]. The appendices of this study illustrate how loss aversion may influence TTO utilities under this normalisation.

For WTD health states, a different approach is needed to incorporate loss aversion. Lipman, Attema and Versteegh [33] developed two corrections to achieve this. The first correction assumes the reference point such that both sides of the WTD indifference elicitation can be seen as losses (i.e., 10 years in *Q* and 10– *T* years in FH). The second correction is such that respondents experience a loss in lifetime in (*T*,* FH*) and a gain in lifetime in *Q* (which is considered negative since it is a WTD health state). In this study, we will use this second approach, as the first approach is not affected by loss aversion. The applied evaluation of indifferences for lead-time TTO (as in Eq. 5) is shown in Eq. [8].

8 $$\:U\left(Q\right)=\frac{\lambda\:\left(L\right(T)-L(10\left)\right)}{1-L\left(10\right)}$$

## Methods

### Data collection

This study consisted of individual video interviews (conducted by the first author) with 100 adult participants from the UK that were recruited through research platform Prolific, which facilitates (online) research participation and is mostly used for studies that rely on surveys or collect data in a similar way. The respondents were awarded a participation fee of around ₤8.00. The interviews on average took 45 to 50 min. The sample size was based on what was feasible for this study and is comparable to other studies (such as Kreimeier et al., [22])^1^. Before recruitment started, ethical approval was obtained via the Erasmus School of Health Policy and Management Research Ethics Review Committee. Participants were asked to sign an informed consent form before proceeding with the interview. Throughout the interview, the interviewer explained the tasks and answered any questions that arose. The interviewer shared her screen with respondents, who stated their answers and preferences verbatim. Since the skills of the interviewer affect the results [40], the interviewer completed training and multiple practice interviews before data collection started.

#### Study design

The interviews were organised in two blocks– a cTTO block and a loss aversion (LA) block. Each respondent completed both blocks from a child perspective (defined as a 10-year-old as is usual in EQ-5D-Y-3L valuation) and from an adult perspective (defined as the respondent themselves). To minimize biases in the responses, the tasks were randomised in three different ways: (1) the order of the blocks was randomised, (2) the order of the child and adult perspective within blocks was randomised, and (3) the order of the six health states that individuals valued within the cTTO block was randomised. The health states that were used differ in severity and were selected from a set of states included in the study by Kreimeier et al. (2018) [22]: 11121, 22222, 23321, 32211, 33323, and 33333. These health states intentionally cover all levels of each dimension.

#### Interview procedure: cTTO block

**Fig. 1:**
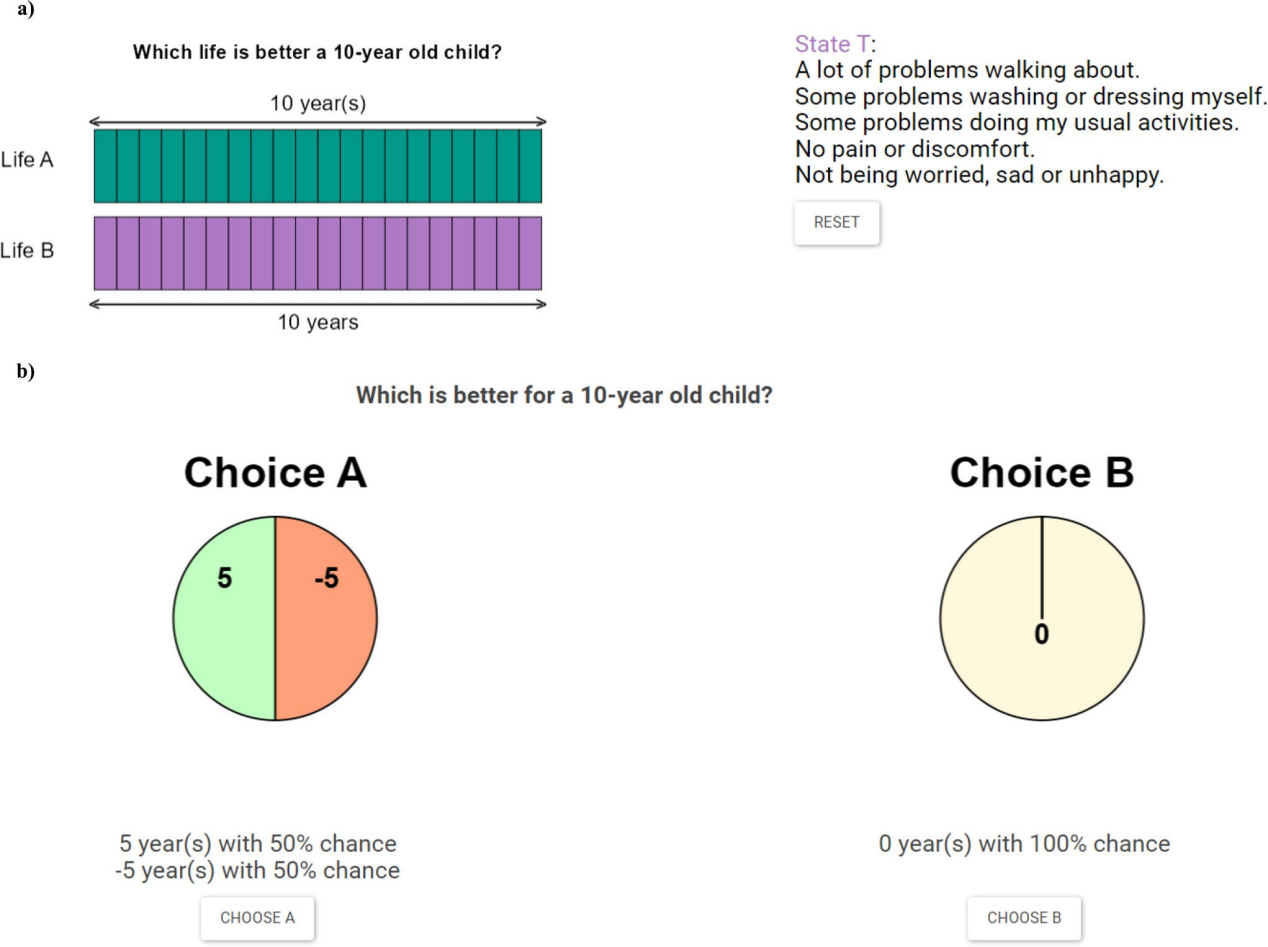
Visualizations of the interview software. **a**) Example of a TTO task from the child perspective, in which life A entails living in full health and life B means life in health state T. **b**) Example of a loss aversion task from the child perspective

Before the tasks started, respondents were asked about their age and gender. They were also asked to fill out the EQ-5D-Y-3L. Additionally, they scored their own health on a scale of 0 to 100 (a visual analog scale). In the cTTO block, respondents completed a ‘wheelchair’ warm-up task based on the procedure outlined in Stolk et al. [41] before moving on to the actual tasks (see appendix I). The cTTO tasks included a ‘sorting’ question in which respondents were asked to choose between living in the impaired health state for 10 years or living in full health for 0 years. The remainder of the task was based on a bisection choice procedure for eliciting indifferences; respondents would be asked to imagine living in a described health state, after which they were asked to choose between different scenarios to elicit the indifference $\:(10,Q)\:\sim\:(T,\:FH)$. The choice started with equal life durations for both scenarios after which the remaining possible interval was continuously cut in half until the respondent indicated to be indifferent between the two scenarios. This meant that respondents were first asked to choose between $\:(10,Q)$ and $\:(10,FH)$. The bisection procedure dictates that choosing $\:(10,FH)$ would be followed by deciding between $\:(5,Q)$ and $\:(10,FH)$, after which the remaining life expectancy was changed again for the following question (i.e., choosing $\:(5,Q)$ leads to $\:(2.5,Q)$ versus$\:\:\left(10,FH\right)$, and choosing $\:(10,FH)$ leads to $\:(7.5,Q)$ versus$\:\:\left(10,FH\right)$). This bisection choice procedure yielded indifferences at a precision of 0.5 years. If a participant indicated that they prefer death over living in the health state in question, the task switched to a lead-time TTO, eliciting indifference $\:(10,FH;10,Q)\:\sim\:(T,\:FH)$. An example of a cTTO task is visualised in Fig. 1a.

#### Interview procedure: loss aversion block

The non-parametric method was used to measure loss aversion [42]. This method can be used to estimate all parameters in prospect theory, but in this study an abbreviated version is used to estimate loss aversion. The method is based on the elicitation of three chained indifferences: (1) a mixed prospect involving both gains and losses, followed by (2) a certainty equivalent in the loss domain $\:({x}_{1}^{-}$), and (3) a certainty equivalent in the gain domain ($\:{x}_{1}^{+})$ [42]. These elicitations can then be used to estimate loss aversion by combining the utilities for the loss and the gain [43]. In this study, individual-level, perspective-dependent loss aversion coefficients were calculated– with *λc* indicating a loss aversion coefficient for the child perspective and *λa* indicating a loss aversion coefficient for the adult perspective. An example of the elicitations can be found in Table 1 [33]. For the first indifference task, $\:G$ and $\:r$ were determined a priori (they are set by the experimenter) such that a value for $\:\mathcal{L}$ can be elicited. Consequently, that value is used as input for the second indifference task, in which the value for loss $\:{x}_{1}^{-}$ is elicited. In the final indifference task, $\:G$ is used again to elicit a value for gain $\:{x}_{1}^{+}$. Finally, using $\:{x}_{1}^{-}$ and $\:{x}_{1}^{+}$, the loss aversion coefficient $\:\lambda\:$ can be calculated.

**Table 1:** An example of the non-parametric method in which$\:{\varvec{x}}_{0.5}\varvec{y}$denotes a gamble resulting in X with a probability 0.5 and y otherwise

	General notation	Goal	Example
Indifference 1: Mixed prospect	$\:{G}_{0.5}\mathcal{L}\:\sim\:r$	Eliciting$\:\mathcal{L}$	$\:{5}_{0.5}-3\:\sim\:0$
Indifference 2: Certainty equivalence– losses	$\:{\mathcal{L}}_{0.5}r\:\sim\:{x}_{1}^{-}$	Eliciting$\:{x}_{1}^{-}$	$\:{-3}_{0.5}0\:\sim-1$
Indifference 3: Certainty equivalence– gains	$\:{G}_{0.5}r\:\sim\:{x}_{1}^{+}$	Eliciting$\:{x}_{1}^{+}$	$\:{5}_{0.5}0\:\sim\:2$
Köbberling & Wakker (2005)	$\:\lambda\:=\:\frac{{x}_{1}^{+}}{{-x}_{1}^{-}}$	Loss aversion coefficient	$\:\lambda\:=\:\frac{2}{-(-1)}=2$

Note. Reprinted and adapted from Lipman, S. A., Attema, A. E. & Versteegh, M. M. (2022). Correcting for discounting and loss aversion in composite time trade-off. *Health Economics*,* 31* [8], 1633–1648. DOI: 10.1002/hec.4529

The reference-point ($\:r)$ is used to compare gains and losses to. As stated above Eq. [7], the reference-point assumed to be of relevance to TTO valuation of EQ-5D instruments is usually 10 years. Several studies have explored other reference-points in TTO and other health preference elicitations [34, 44, 45], though in this study we stick with the commonly used reference-point of 10 years. Respondents were informed that all outcomes in the non-parametric method were added to or subtracted from those 10 years. $\:G$ was set to 5 such that there was a probability of 0.5 that remaining life expectancy increased by 5 years. That is, the first indifference in this study was designed to derive what amount of life years lost $\:\mathcal{L}$ would make respondents indifferent between gambling on the 50/50 chance of living 15 extra years ($\:10+G$) or living $\:10-\mathcal{L}$ life years versus living 10 life years with no extra life years gained (10 + *r*, in which *r* = 0).

In the loss aversion block, tasks were completed to measure λ (designed as in Lipman, Attema and Versteegh [33]). The warm-up task in this block asked respondents to imagine that they had 10 more years left to live, after which they passed away immediately and painlessly. They were offered two options: option A represented a risky scenario that yielded a 50% chance of gaining an additional 8 years (on top of the promised 10 years) and a 50% chance of losing 8 years (of the promised 10 years), option B meant that respondents gained or lost 0 life years (i.e., they lived the promised 10 years with 100% certainty).

The interviews were organised as follows: the interviewer guided participants through the different tasks, which were visualized using R Shiny. Visualizations can be found in Fig. 1, and the entire interview interface can be clicked through here: https://referencepoints.shinyapps.io/LossAvarsion/ After the tasks ended, respondents were asked about their religious beliefs, their parental status, and their educational background.

### Data analysis

All data analyses were performed in R. A data repository can be found under DOI: 10.25397/eur.28706093. Before starting the main analyses of the results, several data quality controls were performed. Firstly, a descriptive analysis was carried out to get an overview of the respondents’ characteristics to ensure that the sample shows a mix in terms of age, gender, level of education and parental status. In addition, the randomisation of perspectives was assessed to review the risk of bias due to the order in which the tasks were completed. Secondly, given that concerns have been raised about TTO utility data in some publications [40, 46], following earlier work [24], the data quality was examined and compared between the perspectives using chi-square tests. This was done by looking at the number of non-trading responses (utility = 1), all-in trading responses (utility = -1), zero responses (utility = 0) and the frequency of unique observations being less than 50% (i.e., fewer than 3 unique values per perspective). Furthermore, the number of respondents without negative values and without 0.5-year increments (as this would suggest low precision) were assessed. Lastly, it was checked how many of the responses showed a violation of weak dominance (e.g., utility of health state 22222 ≤ 33333) and how many responses were a strict dominance violation (e.g., utility of health state 22222 < 33333). The health states included in this study create 15 possible pairs to test dominance violations on^2^. Note that although we refer to these analyses as data quality controls, the occurrence of the respondent patterns summarized above need not imply low quality decision-making (e.g., utilities of 1 may occur due to low task engagement, but also as a result of deliberate and well-reasoned decisions not to sacrifice life duration).

#### Loss aversion correction

The results of the loss aversion elicitations were used to calculate two different loss aversion coefficients: *λa* and *λc*. This was done using the methods described in the loss aversion block in Sect. 3. cTTO utilities were rescaled and corrected for loss aversion using Eqs. [7] and [8]. In this correction, *λa* was used for the adult cTTO utilities, and *λc* was used for the child cTTO utilities. The utilities that result from this correction for loss aversion will henceforth be called ‘corrected utilities’. The utilities that result from the interviews and have not been corrected, will be called ‘uncorrected utilities’ and are based on Eqs. [4] and [6].

#### Analyses of cTTO utilities

We first report descriptive results of the cTTO utilities, both before and after correction. T-tests were performed to see if any of the health states showed a statistically significant difference between adult and child perspectives. The results of these tests are reported alongside the mean uncorrected and corrected utilities. In addition to the means, the differences between the perspectives were calculated per health state. This was done by calculating individual-level difference scores ($\:\varDelta\:{\prime\:}s\:$) based on Eq. [9], and aggregating the individual absolute differences.

9 $$\:\varDelta\:c\text{T}\text{T}\text{O}\:=\:{cTTO}_{A,i}-{cTTO}_{C,i}$$

Here cTTO_A, i_ denotes the adult cTTO utility for respondent i, and cTTO_C, i_ denotes the child cTTO utility for respondent i.

#### Regression analyses

Two types of linear mixed effect models were constructed: models with the uncorrected utilities and models with the corrected utilities. The models included fixed effects for the perspective (with adult as the reference category) and for the health states (with 11121 as a reference category) and random effects for the subjects. Furthermore, they also included fixed effects for gender (female being the reference category) and parental status (with no children as the reference category). To further explore the role of loss aversion, separate analyses were conducted for BTD and WTD states. In the latter, 33333 served as a reference category for the health states.

## Results

### Characteristics of the sample and data quality

An overview of the characteristics of the respondents can be found in Table 2. The sample consists of a mix of gender and parental status. The respondents were predominantly highly educated. The mean age of the respondents is 39 (12.2) years (SD), with the youngest respondent being 20 and the oldest being 76 years old. Due to a technical error, the age of one respondent is missing in the data. Respondents were generally healthy– the most frequently self-reported health states were 11111 and 11112.

**Table 2:** Descriptive statistics of respondents (*n* = 100)

Variable	Stats / Values	Frequency (%)
First perspective^a^	A 10-year-old child Yourself	52 (52.0%) 48 (48.0%)
Gender	Female Male Non-binary	58 (58.0%) 41 (41.0%) 1 (1.0%)
Education^b^	College Graduate or professional or University Bachelor’s degree Secondary school Vocational or similar	17 (17.0%) 77 (77.0%) 4 (4.0%) 2 (2.0%)
Parental status	No Yes	51 (51.0%) 49 (49.0%)
*Self-reported health (EQ VAS)*	*Minimum* *Median* *Maximum* *Mean*	*25* *80* *100* *78 (14.6 SD)*

^a^The first perspective that the respondent had to complete the valuation tasks from

^b^The highest level of education the respondent completed

**Fig. 2:**
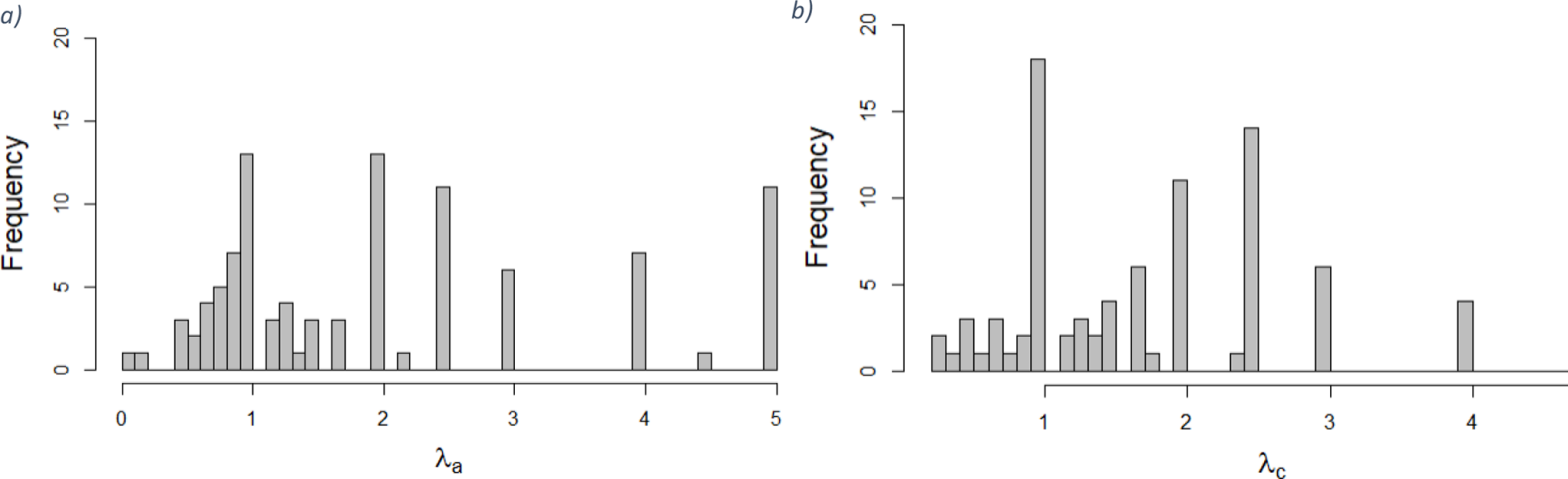
Individual-level loss aversion coefficients (λ) from the adult ($\lambda_{a}$) and child (λ_C_) perspective

Table 3 shows an overview of the data quality checks that were performed. The quality checks show some interesting differences between the perspectives, albeit they are not all statistically significant. Looking at the most extreme trading options, there were more non-traders and less all-in traders for the child perspective. In line with the histograms shown in Fig. 2; Table 3 shows more utilities of zero for the child perspective than for the adult perspective. Based on these checks, the data quality seems to be reasonable, though the number of strict violations of dominance in the sample is relatively high (8.86% for the adult and 7.65% for the child perspective). Part of the weak violations of dominance are explained by all-in trading for health states less severe than 33333. That is, 26 occurrences in the adult and 21 in the child perspective were the result of health states less severe than 33333 being valued at the lowest possible utility of -1, leaving no room for dominance of health state 33333.

**Table 3:** Data quality per perspective

Response pattern	Adult perspective	Child perspective
Non-trading responses (U = 1) *(n = 598)*	21	24
**All-in trading responses (U=-1)** ***(n = 598)***	**43**	**26**
**Zero responses (U = 0)** (*n* = 598)	**12**	**26**
Fewer than 3 out of 6 unique observations^a^ (*n* = 100)	1	7
Respondents without negative utilities (*n* = 100)	47	53
Respondents without 0.5-year increments (*n* = 100)	5	8
**Weak violation of dominance (e.g., 22222 ≤ 33333) (** *n* ** = 1,490)**	**269**	**327**
Strict dominance violation (e.g., 22222 < 33333) (*n* = 1, 490)	130	111

^a^Respondents that gave the same value to all health states, or that had only 2 unique valuations

Bold-faced analyses indicate that chi-square tests were significant (*p* < 0.05)

### Correcting for loss aversion

The distributions of the loss aversion coefficients were skewed with some outliers at very high values due to some respondents’ unwillingness to take any sort of gamble when it comes to life years (e.g., loss aversion coefficients of 25). Such large coefficients for loss aversion would strongly affect TTO utilities after correction. To improve the data analysis and minimize the effect of these outliers, they were altered by capping the loss aversion coefficients at a maximum value of 5 (specified as $\:\lambda\:$ ≤ 5). This led to 3 values being censored for the adult perspective, and 9 for the child perspective. It is worth noting that the highest coefficient in the child perspective was almost three times greater than the highest value for the adult perspective. Figure 2 reports the distribution of the coefficients after capping them. For the sake of completeness, the same analyses were performed without censoring the data. These analyses can be found in the appendices of this paper– the conclusions of all of the following analyses remain largely unchanged.

 Without censoring, there seems to be a difference in loss aversion between the adult and the child perspective. When removing outliers by censoring the loss aversion data, this difference disappears. The standard deviation shows a strong decrease after censoring the child perspective loss aversion coefficients, which is in line with the occurrence of more outliers in this perspective and the difference in magnitude of the outliers between the perspectives. In any case, the difference between perspectives is not statistically significant (*p* = 0.196 with no censoring, *p* = 0.751 with $\:\lambda\:$ ≤ 5, based on T-tests). Table 4 shows the difference between perspectives in the TTO utilities relative to the individual-level loss aversion coefficients (i.e., it shows (in)consistency within respondents). A chi-square test indicates no statistically significant difference between the categories of loss aversion coefficients and the average difference between perspectives in TTO utilities for each respondent.

**Table 4:** Consistency between individual loss aversion coefficients and differences between perspectives in TTO utilities

	Number of respondents	Adult utility > child utility	Adult utility = child utility	Child utility > adult utility
λa > λc (*N* = 234)	39	71 *(30.34%)*	60 *(25.64%)*	103 *(44.02%)*
λa = λc (*N* = 72)	12	23 *(31.94%)*	29 *(40.28%)*	32 *(44.44%)*
λa < λc (*N* = 294)	49	82 *(27.89%)*	62 *(21.09%)*	199 *(67.69%)*

### cTTO utilities

Table 5 shows the mean uncorrected and the mean corrected utilities of each health state, specified for both perspectives. For the uncorrected utilities, health states 32211, 23321, and 33323 show a statistically significant difference between the adult and the child perspective, with the child perspective yielding higher utility. For the corrected utilities, health state 23321 is significantly different between perspectives. Overall, some trends can be seen in the utilities. Both before and after correcting for loss aversion with $\:\lambda\:$ ≤ 5, most health states show higher utility from a child perspective than from an adult perspective. After correction, all utilities decrease. Table 5 also shows that the absolute differences (Δ) between perspectives per health state increase after correction. Combined with the increasing standard deviations, this suggests that correction decreases mean cTTO utility estimates and increases the heterogeneity of these estimates.

**Table 5:** Mean (standard deviations) cTTO utilities for all States per perspective and the differences between perspectives with data censoring (LA ≤ 5)

	Uncorrected utilities		Corrected utilities		Differences between perspectives	
*State*	cTTO-A	cTTO-C	cTTO-A	cTTO-C	Δ Uncorrected	Δ Corrected
*11121*	0.86 (0.16)	0.86 (0.16)	0.8 (0.19)	0.79 (0.19)	-0.002	0.002
*32211*	0.63 (0.36)**	0.71 (0.32)**	0.39 (1.22)	0.57 (0.55)	-0.086	-0.186
*22222*	0.61 (0.36)	0.62 (0.36)	0.39 (1.13)	0.44 (0.71)	-0.011	-0.05
*23321*	0.46 (0.53)**	0.59 (0.43)**	0.03 (1.69)*	0.34 (0.93)*	-0.132	-0.312
*33323*	0.03 (0.62)*	0.14 (0.61)*	-0.79 (2.13)	-0.56 (1.68)	-0.113	-0.217
*33333*	-0.18 (0.62)	-0.1 (0.59)	-1.62 (2.53)	-1.29 (2.33)	-0.082	-0.331

* Indicates that the within-subject difference between the adult and child valuations was significant (T-test, * = *p* < 0.05, ** = *p* < 0.01, *** = *p* < 0.001). Statistical significance with Wilcoxon signed-rank test: uncorrected, 32211***, 23321***, 33323*, corrected: 32211***, 23321**

### Regression results

Table 6 shows the first two linear mixed effect models. The models are based on both uncorrected and corrected utilities. Model 1 was computed without including any demographic characteristics. Model 2 includes gender, parental status, highest completed level of education (for which college is the reference category) and the order of the perspectives during the tasks as additional variables. Both models use state 11121 as a reference category for the state dummies. Both model 1 and 2 report a statistically significant difference between the child and adult perspective at the 99% significance level for the uncorrected utilities and 95% for the corrected utilities. In line with the results in Table 5, utilities are higher for the child perspective. Adding demographic characteristics to the model slightly increases the AIC and therefore marginally worsens the model’s explanatory power. None of the reported characteristics significantly impact cTTO utilities.

**Table 6:** Linear mixed models of cTTO utilities for health States from the adult and child perspective with and without demographics (*n* = 1169)

	Model 1		Model 2	
	Uncorrected utilities	Corrected utilities	Uncorrected utilities	Corrected utilities
*term*	*Estimate (SE)*	*Estimate (SE)*	*Estimate (SE)*	*Estimate (SE)*
*(Intercept)*	0.83 (0.04) ***	0.70 (0.12) ***	0.89 (0.13) ***	0.68 (0.34) *
*State 32211*	-0.20 (0.03) ***	-0.32 (0.13) *	-0.20 (0.04) ***	-0.32 (0.13) *
*State 22222*	-0.25 (0.03) ***	-0.38 (0.13) **	-0.25 (0.04) ***	-0.38 (0.13) **
*State 23321*	-0.34 (0.04) ***	-0.61 (0.13) ***	-0.34 (0.04) ***	-0.61 (0.13) ***
*State 33323*	-0.77 (0.03) ***	-1.47 (0.13) ***	-0.78 (0.04) ***	-1.47 (0.13) ***
*State 33333*	-1.00 (0.03) ***	-2.25 (0.13) ***	-1.01 (0.04) ***	-2.25 (0.13) ***
*Child perspective*	0.08 (0.02) ***	0.18 (0.08) *	0.07 (0.02) ***	0.18 (0.08) *
*Gender: Non-male*	-	-	0.01 (0.07)	0.003 (0.17)
*Kids: yes*	-	-	0.05 (0.06)	0.09 (0.16)
*Secondary school*	-	-	0.12 (0.17)	0.57 (0.45)
*Vocational or similar*	-	-	0.23 (0.24)	0.70 (0.61)
*University Bachelor’s degree*	-	-	-0.03 (0.09)	0.10 (0.23)
*Graduate or professional degree*	-	-	-0.06 (0.1)	0.01 (0.24)
*Order of perspectives*	-	-	-0.05 (0.07)	-0.08 (0.17)
AIC	1167.274	4227.316	1197.9	4245.1

***p < 0.001,**p < 0.01,*p < 0.05

Table 7 Shows two linear mixed effect models that separate BTD and WTD cTTO utilities. In the WTD model, health state 33333 serves as a reference category for the health state dummies. Model 3 (BTD only) shows that the utilities decrease in accordance with health state severity. In model 3, both before and after correction, the effect of perspective significantly impacts cTTO utilities (i.e., *p* < 0.001). Model 4 (WTD only) shows that the effect of perspective on cTTO utilities is no longer statistically significant after correcting for loss aversion. Furthermore, model 4 shows that correction causes a strong increase in WTD utility values, which explains the steep increase in absolute differences between perspectives described in Table 5. Note that none of the fixed effects for health states are significant, indicating that differences with the utility of state 33333 are not significant. In addition, the intercept and the standard errors are significantly higher for the WTD health states compared to the BTD health states.

**Table 7:** Linear mixed models that separate better than dead (BTD) and worse than dead (WTD) utilities for six different health states from the adult and child perspective

	Model 3: BTD only (*n* = 1000)		Model 4: WTD only (*n* = 196)	
	Uncorrected utilities	Corrected utilities	Uncorrected utilities	Corrected utilities
*term*	Estimate (SE)	Estimate (SE)	Estimate (SE)	Estimate (SE)
*(Intercept)*	0.84 (0.02) ***	0.78 (0.02) ***	-0.72 (0.04) ***	-3.57 (0.3) ***
*State 32211*	-0.15 (0.02) ***	-0.17 (0.02) ***	0.01 (0.07)	0.13 (0.64)
*State 22222*	-0.20 (0.02) ***	-0.22 (0.02) ***	0.08 (0.07)	-0.08 (0.6)
*State 23321*	-0.22 (0.02) ***	-0.25 (0.02) ***	0.05 (0.05)	0.16 (0.43)
*State 33323*	-0.44 (0.02) ***	-0.47 (0.02) ***	0.01 (0.03)	0.12 (0.25)
*State 33333*	-0.53 (0.02) ***	-0.55 (0.02) ***	-	-
*Child perspective*	0.04 (0.01) ***	0.04 (0.01) **	0.09 (0.03) **	0.46 (0.25)
AIC	-399.4	-285.9	19.7095	831.3

***p < 0.001, **p < 0.01, *p < 0.05

## Discussion

The main motivation of this study was to explore the extent to which loss aversion could explain differences in cTTO utilities elicited with adult and child perspectives. Overall, the results of this study suggest that differences in the degree of loss aversion for adults versus children do not explain the differences in cTTO utilities between the perspectives. When looking at the utilities per health state, we replicate results shown in earlier work. That is, there seem to be differences between utilities elicited from an adult and from a child perspective [15, 22–24], with a similar number of health states yielding higher utilities for the child perspective compared to the adult perspective [24]. Our work shows a trend for children’s health states to yield higher utility than adult health states. The magnitude of the differences differs between studies [22–24]. Furthermore, it is worth noting that the models on the WTD data show that utility does not differ significantly between 33333 and other WTD states, replicating earlier work that questions the sensitivity of cTTO for WTD health states [47, 48].

We observed some differences in loss aversion when comparing adult and child perspectives. Loss aversion showed large heterogeneity for children vis-à-vis adults, but there was no statistically significant difference– the individual-level mean loss aversion coefficient was 2.09 (1.43 SD) for the adult and 2.2 (1.45 SD) for the child perspective. These coefficients are in line with a meta-analysis by Brown et al. [30]. The absence of a mean difference suggests that loss aversion is a consistent phenomenon that is independent of perspective, which is in line with earlier research into loss aversion by Lipman, Brouwer and Attema [49], who found loss aversion to, on average, be independent of the severity of the health states considered. Interestingly, studies into loss aversion for monetary outcomes find contrasting results. For instance, Mengarelli et al. [35] showed that loss aversion reduced when making a monetary choice for another person compared to making the choice for themselves. When deciding whether to enter lotteries, people seem to be less loss averse when it comes to making financial decisions for others if the decision bears no consequences for themselves [36]. Polman [38] drew the same conclusion– people are less loss averse when making decisions for others compared to making decisions for themselves– when it comes to riskless choice, gambling, and social factors. The absence of the same difference for health-related questions suggests that health may be an exception to this behaviour. Several researchers find evidence that supports this suggestion. For example, Pachur, Hertwig and Wolkewitz [50] indeed found that people have different preferences for ‘affect-poor’ (e.g., monetary) and ‘affect-rich’ (e.g., health-related or amenity-related) choice tasks. Future research should be focused on further investigating whether health is unique when it comes this kind of choice behaviour– for example, by exploring within-subject differences in choice behaviour regarding monetary matters versus health-related decisions.

The analyses on correcting for loss aversion showed that, as reported in previous work [33], correction decreased utilities. Our work adds to this literature that this downward effect of correction occurs in both perspectives, and (particularly for WTD states) increases heterogeneity. The linear mixed effect models in this paper demonstrate that loss aversion does not explain the difference in cTTO utilities between the adult and the child perspective. In all models, the corrections are accompanied by increased error– the mean absolute differences between perspectives and the utilities’ standard deviations/errors tend to increase– due to uncertainty in the loss aversion estimates.

As far as we know, no other studies on health-related loss aversion from the adult and child perspective have been done, which complicates comparison with existing literature. Nevertheless, several qualitative studies found people to be reluctant to trade-off life years for children [11, 12]. This study was motivated by the notion that (the underlying causes for) this reluctance may correlate with people showing more loss aversion for children, since the same underlying principles play a role in loss aversion tasks. Nevertheless, our study provides no evidence in favour of this reasoning, as we find no consistent evidence to suggest that differences between the perspectives disappear after correction.

### Limitations

This study has several limitations. First of all, the respondents in this study tended to be higher educated, which has consequences for the representativeness of the study sample. Second, the number of violations of dominance was relatively high, potentially due to recruiting people for online rather than in-person interviews. Third, the most apparent limitation is the singular focus on loss aversion and consequently ignoring potential other mechanisms. For example, a limitation that might explain our null result is the assumption of utility being linear. To illustrate: Lang, Attema & Lipman [27] found that the difference between the adult and the child perspective can be partly explained by differences in time preference (i.e., nonlinearity of utility)– they state that the adult-child differences disappear when correcting cTTO utilities for time preference. Furthermore, the need to censor the loss aversion coefficients might indicate that the loss aversion task is influenced by other behaviours– for instance by (un)willingness to gamble– hence affecting its ability to explain or capture unwillingness to trade-off life duration. Further research that takes into account both loss aversion and utility curvature could provide a better understanding of the effects of loss aversion on cTTO utilities.

Another limitation could be the difference in the length of the scale that utilities are reported from before and after correction. Before correction the utilities lie between − 1 and 1, whereas after correction, the assumption of linearity is dropped and the lower limit of -1 disappears. As a result, the corrected utilities show outliers far below − 1. As pointed out in existing literature, these exceptionally low utility scores may pose issues when using them in cost-effectiveness analyses [51]. However, there seems to be little to no basis for using a lower limit of -1 [51], since this limit is arbitrary and is an artefact of the valuation method used [33]. Though transforming utility data to fit this limit simplifies data analysis, it may also mean that, when looking at life duration, utilities of BTD and WTD states can no longer be compared on the same scale, which complicates their use for QALY computation and ultimately cost-effectiveness analyses.

A final limitation of this study is the reference-point that is used in the corrections. It appears individual’s expectation about length of life in particular could serve as a reference-point [34]. The effect associated with such subjective life expectancies is that people are reluctant to trade-off years that are below the number of years they thought they had left to live. For example, if people expect to live for another 50 years and you ask them to trade-off 10 years, people are more reluctant to give up life years than if you would ask them to trade-off 70 years. For the child perspective, this may mean that the life expectancy people expect a child to have influences the way they value health states on behalf of children. The correction for loss aversion in this study is based on a reference-point of 10 years. Existing research has found mixed results; some studies found that the reference-point does not have any effect [52], others find that it does [44, 53]. If subjective life expectancy would serve as the reference-point instead of the assumed 10 years, that would mean that the corrections that are carried out in this study are incorrect.

### Conclusions

Overall, this study shows that loss aversion cannot explain perspective-dependent differences in utilities elicited with adult and child perspectives. In fact, our findings somewhat caution against correcting for (only) loss aversion: although loss aversion has been argued to cause bias in cTTO, the increased error that accompanies correction of cTTO utilities for loss aversion seems undesirable. At face value our results suggest that correcting bias related to loss aversion may even increase differences between utilities elicited from adult and child perspectives, potentially exacerbating the effects these differences could have on ICERs. That is, when utilities elicited with adult EQ-5D instruments and adult perspectives, are compared to those elicited with EQ-5D-Y-3L instrument, the perspective used in the latter instrument may influence the outcome of the comparison. Whether or not this is desirable ultimately depends on the causes of these differences. For example, they could be desirable if they reflect true differences in (the perception of) health state severity (as discussed by Devlin et al. [54]), and/or the potential effects on reimbursement decision-making are in line with societal preferences (something that might be improved using equity weights, as suggested by Attema, Lang & Lipman [55]). Therefore, although our study provides no evidence for loss aversion as one of these explanations, further research into other causes of differences between the adult and the child perspective and their potential consequences for reimbursement decisions remains relevant. In addition, we would recommend repeating this study with a larger sample size and/or a larger number of health states.

## Electronic supplementary material

Below is the link to the electronic supplementary material.

Supplementary Material 1

## Footnotes

1 Note that EQ-5D value sets (e.g. Versteegh et al., 2016) are typically also elicited with designs in which each included health state is valued by approximately 100 participants.

2 The potential dominance violations are (denoted as state1 < state2 meaning utility of state 1 lower than that of state 2): 11121 < 32211, 11121 < 22222, 11121 < 33323, 11121 < 33333, 11121 < 23321, 32211 < 22222, 32211 < 33323, 32211 < 33333, 32211 < 23321, 22222 < 33323, 22222 < 33333, 22222 < 23321, 33323 < 33333, 23321 < 33323, 23321 < 33333.
