# Supplementary material for: Loss aversion in EQ-5D-Y-3L: does it explain differences in willingness to trade-off life years in adults and children?
Source: Eur J Health Econ. 2025 Apr 12;26(8):1369–81. doi: 10.1007/s10198-025-01775-6 (PMC12572096; doi:10.1007/s10198-025-01775-6)
Supplement: Supplementary file 1 — Supplementary Material 1 [file 10198_2025_1775_MOESM1_ESM.docx]

# Appendices

## Appendix I – Explanation of wheelchair warm-up

Before respondents were asked to complete the TTO tasks, they were given a warm-up task, as outlined in Stolk et al. (2019). This task is designed to show the range of possible answers in the TTO tasks. Respondents were asked to compare full health to being in a wheelchair. After a utility value was established for being in a wheelchair, there were two scenarios:

*Better than dead example*

If the respondents considered being in a wheelchair worse than being dead, the follow-up question asked them to think of a state of health they consider to be better than dead (e.g., some pain every day). The respondents would then get a second warm-up question based on their example of a state of health that is better than being dead, to familiarize them with the TTO task.

*Worse than dead example*

If the respondents considered being in a wheelchair better than being dead, the follow-up question asked them to think of a state of health they consider to be better than dead. If respondents couldn’t come up with an example, the interviewer would provide them with their own example and explained why they would consider this to be better or worse than being dead. The respondents would then get a second warm-up task that asked them to value the worse than dead health state in a lead-time TTO task, to make sure each respondent is familiar with this task as well.

## Appendix II – Distribution of cTTO data

Histograms were computed to visualize the distribution of the values the respondents assigned to the health states. Part a) of figure 3 shows a histogram of all cTTO utilities that were elicited during the interviews. The utilities are skewed to the right, with higher utilities occurring more frequently. In b) and c), the utilities are reported per perspective.


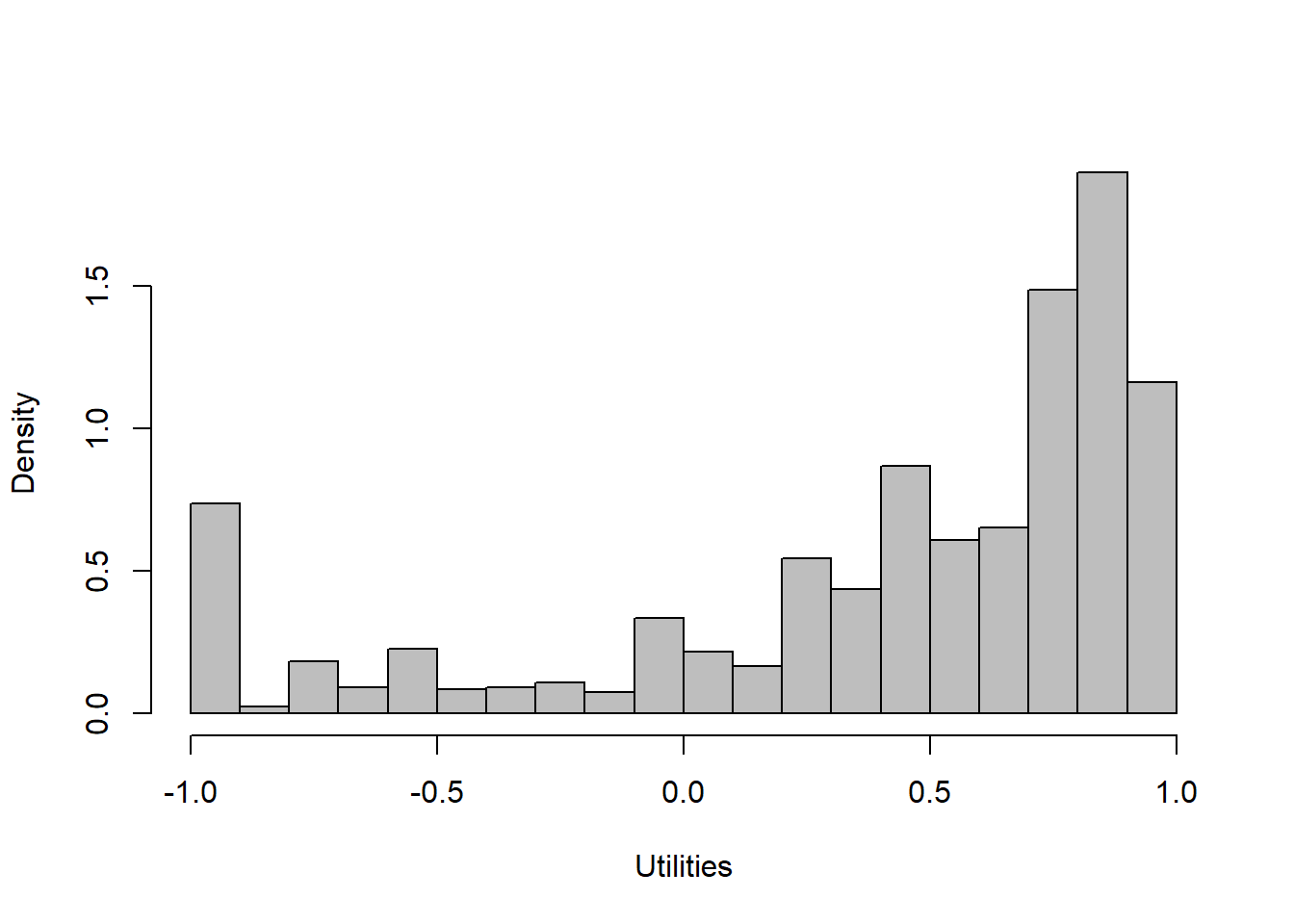

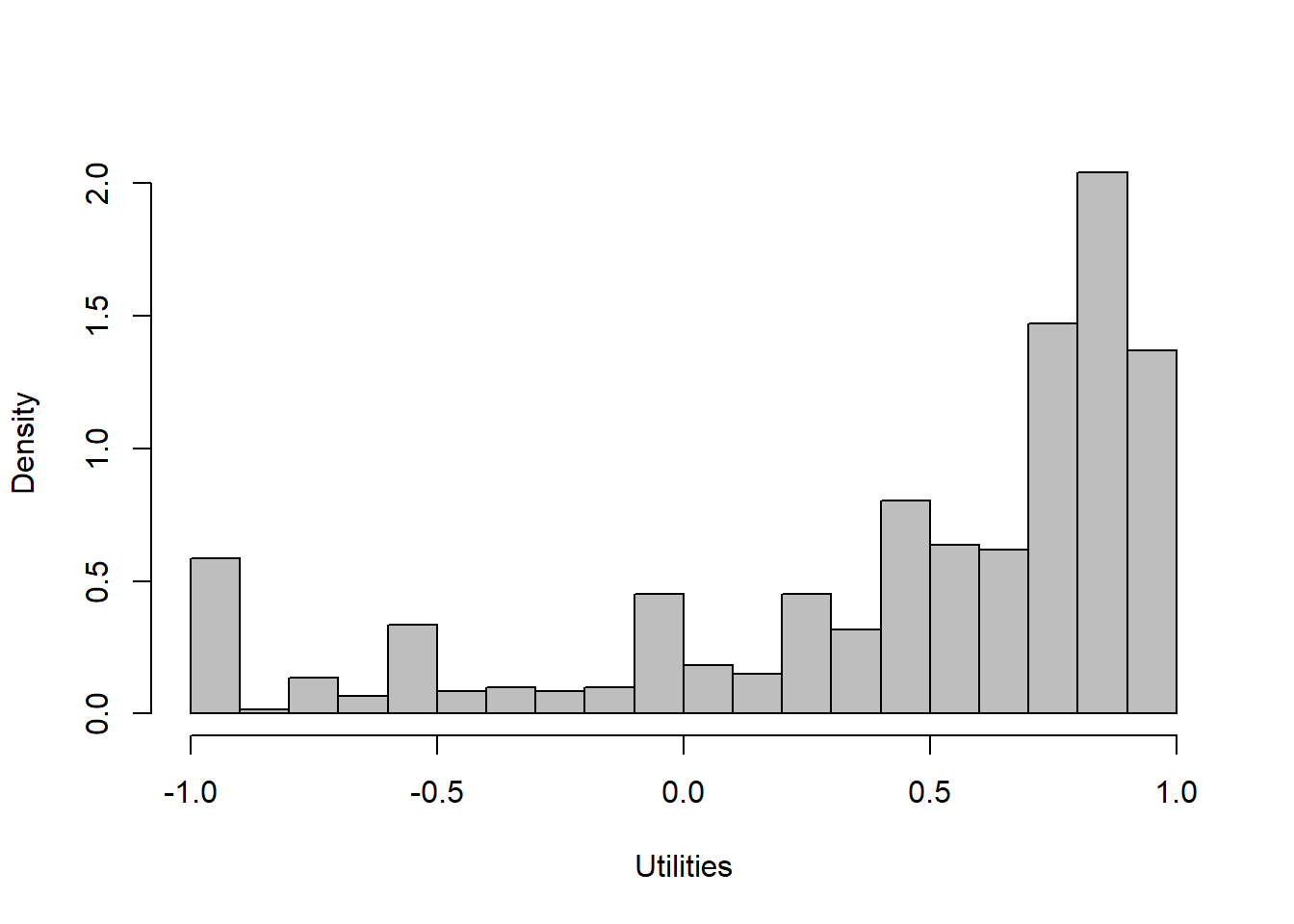

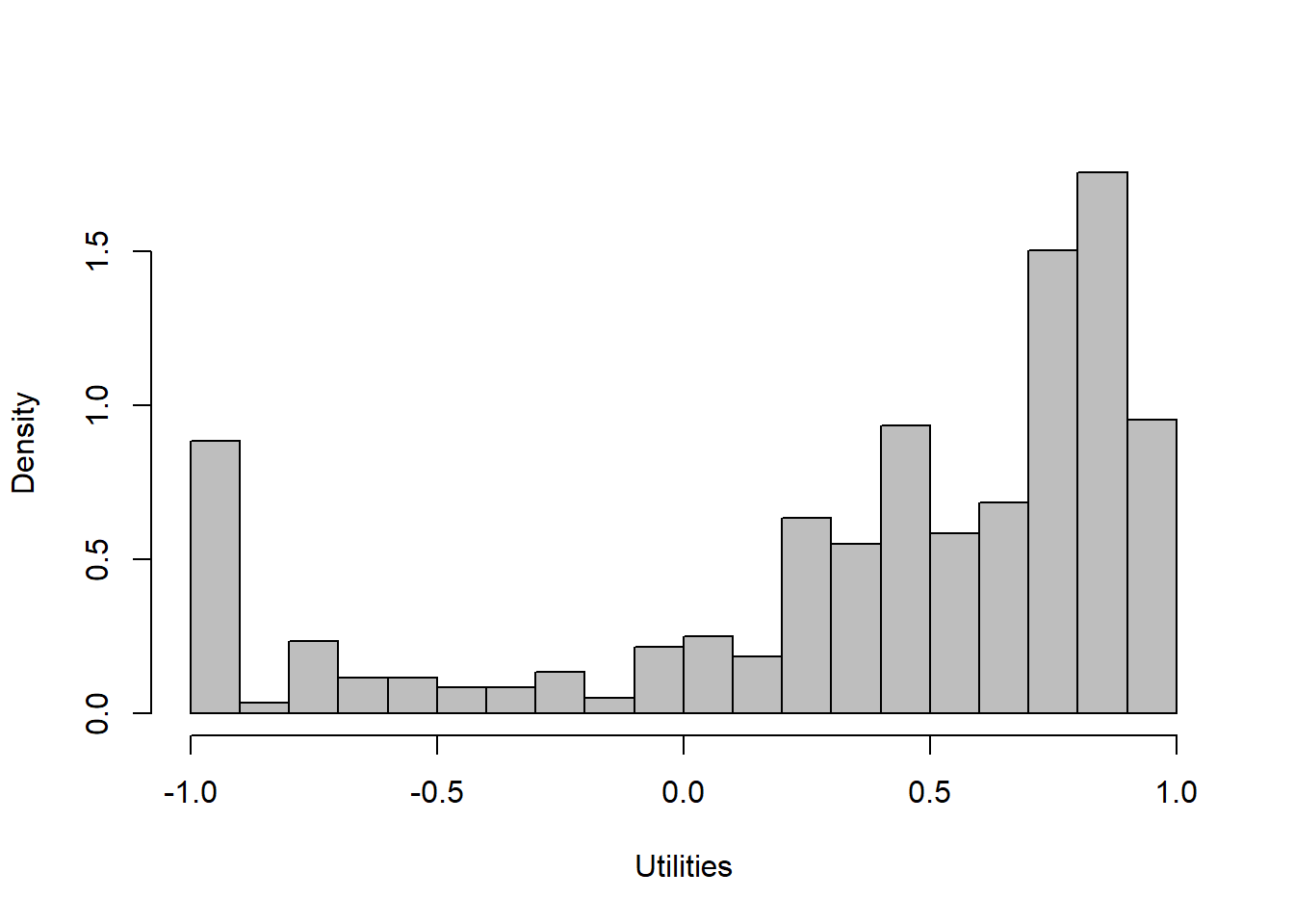


**Fig 3 cTTO utilities**

b) cTTO utilities from the adult perspective

c) cTTO utilities from the child perspective

a) All cTTO utilities

## Appendix III – The effect of loss aversion on TTO utilities

The main motivation for measuring loss aversion in this research paper lies in the hypothesis that differences in utilities between the adult and the child perspective may reflect differences in loss aversion. In other words, people may be more unwilling to give up life duration for children than for adults (relative to gaining life duration), which may be reflected in a higher $\lambda$. If people are more loss averse for children than for adults, this would yield different values for $\lambda$ in equation [7], which in turn would result in different utility values for the same health states. We will illustrate how loss aversion affects TTO by showing its effect on the resulting sacrificed life duration and by showing its effect on utility, given that we assume equal sacrificed life duration to calculate utilities.

**EFFECT OF LOSS AVERSION ON T**

Imagine a person that is not loss averse for adults but is loss averse for children with $\lambda= 2$.

Assume U(Q)=0.5 and $\lambda$ = 1 (i.e., no loss aversion). Using the aforementioned scaling approach and equation (7), this yields:

$0.5=\frac{T/20}{1*(10/20)+\left( 1-1 \right)(T/20)}$ , which with $\lambda$ = 1 gives:

0.5 = (T/20) / 0.5

$T/20= 0.25\to T=5$

Now, we incorporate $\lambda= 2$:

$0.5=\frac{T/20}{2*(10/20)+(1-2)(T/20)}$, which simplifies to

$0.5=\frac{T/20}{1-(T/20)}$,

$0.5=-1 + \frac{1}{1-(T/20)}$

$T/20=1-\frac{1}{0.5+1}$

$T=6.667$

This example illustrates that for this person, for whom loss aversion is stronger for children than for adults, giving up less life years for children (vs. adults) results in the same utility. In other words, different loss aversion coefficients for adults and children lead to different life duration sacrifices for the same health state.

**EFFECT OF LOSS AVERSION ON UTILITY**

Assume T=5 and $\lambda$ = 1 (i.e., no loss aversion as in Eq. 3):

$U(Q)=\frac{\left( 5/20 \right)}{(10/20)}$ = 0.5

Now assume T=5 and $\lambda$ = 2:

$U(Q)=\frac{5/20}{2*(10/20)+(1-2)(5/20)}$

$U(Q)=\frac{0.25}{1-0.25}$ = 0.333

This example shows that differences in loss aversion yield different utilities for children than for adults for the same health state and the same life duration sacrificed. In particular, it shows that correcting for loss aversion generally yields lower utilities.

## Appendix IV – Data without censoring

| **Table 8. Mean (standard deviations) cTTO utilities for all states per perspective and the differences between perspectives without data censoring** | | | | | | |
| --- | --- | --- | --- | --- | --- | --- |
|  | Uncorrected utilities | | Corrected utilities | | Differences between perspectives | |
| *State* | TTO-A | TTO-C | TTO-A | TTO-C | Δ Uncorrected | Δ Corrected |
| *11121* | 0.86 (0.16) | 0.87 (0.16) | 0.79 (0.20) | 0.78 (0.21) | -0.002 | 0.01 |
| *32211* | 0.63 (0.36)** | 0.71 (0.32)** | 0.38 (1.22) | 0.56 (0.55) | -0.086 | -0.179 |
| *22222* | 0.61 (0.36) | 0.62 (0.36) | 0.39 (1.13) | 0.43 (0.71) | -0.011 | -0.043 |
| *23321* | 0.46 (0.53)** | 0.59 (0.43)** | 0.03 (1.69) | 0.33 (0.93) | -0.132 | -0.304 |
| *33323* | 0.03 (0.62)* | 0.14 (0.61)* | -0.79 (2.13) | -0.79 (3.13) | -0.113 | 0.007 |
| *33333* | -0.18 (0.62) | -0.1 (0.59) | -1.63 (2.55) | -1.62 (3.86) | -0.082 | -0.013 |
| ** Indicates that the within-subject difference between the adult and child valuations was significant (T- test, p<0.05).* | | | | | | |

| **Table 9. Linear mixed models of cTTO utilities for health states from the adult and child perspective with and without demographics, with uncensored data (*n=1169*)** | | | | |
| --- | --- | --- | --- | --- |
|  | **Model 1** | | **Model 2** | |
|  | Uncorrected utilities | Corrected utilities | Uncorrected utilities | Corrected utilities |
| *term* | *Estimate (SE)* | *Estimate (SE)* | *Estimate (SE)* | *Estimate (SE)* |
| *(Intercept)* | 0.83 (0.04) *** | 0.74 (0.15) *** | 0.88 (0.13) *** | 0.95 (0.4) * |
| *State 32211* | -0.20 (0.04) *** | -0.32 (0.17) | -0.2 (0.04) *** | -0.32 (0.17) |
| *State 22222* | -0.25 (0.04) *** | -0.38 (0.17) * | -0.25 (0.04) *** | -0.38 (0.17) * |
| *State 23321* | -0.34 (0.04) *** | -0.60 (0.18) *** | -0.34 (0.04) *** | -0.61 (0.18) *** |
| *State 33323* | -0.78 (0.04) *** | -1.58 (0.17) *** | -0.78 (0.04) *** | -1.58 (0.17) *** |
| *State 33333* | -1.01 (0.04) *** | -2.42 (0.17) *** | -1.01 (0.04) *** | -2.42 (0.17) *** |
| *Child perspective* | 0.07 (0.02) *** | 0.09 (0.10) | 0.07 (0.02) *** | 0.09 (0.10) |
| *Gender: Non-male* | - | - | 0.01 (0.07) | -0.06 (0.19) |
| *Kids: yes* | - | - | 0.05 (0.06) | 0.01 (0.19) |
| *Secondary school* | - | - | 0.12 (0.17) | 0.58 (0.51) |
| *Vocational or similar* | - | - | 0.23 (0.24) | 0.74 (0.71) |
| *University Bachelor’s degree* | - | - | -0.03 (0.09) | 0.03 (0.26) |
| *Graduate or professional degree* | - | - | -0.06 (0.1) | 0.04 (0.28) |
| *Order of perspectives* | - | - | -0.05 (0.07) | -0.17 (0.19) |
| AIC | 1167.3 | 4857.6 | 1197.9 | 4873.7 |
| ****p<0.001,**p<0.01,*p<0.05* | | | | |

| **Table 10. Linear mixed models that separate better than dead (BTD) and worse than dead (WTD) utilities for six different health states from the adult and child perspective, with uncensored data** | | | | |  |
| --- | --- | --- | --- | --- | --- |
|  | **Model 3: BTD only *(n=1000)*** | | **Model 4: WTD only *(n=196)*** | |  |
|  | Uncorrected utilities | Corrected utilities | Uncorrected utilities | Corrected utilities |  |
| *term* | Estimate (SE) | Estimate (SE) | Estimate (SE) | Estimate (SE) |  |
| *(Intercept)* | 0.84 (0.02) *** | 0.77 (0.02) *** | -0.72 (0.04) *** | -3.63 (0.45) *** |  |
| *State 32211* | -0.15 (0.02) *** | -0.17 (0.02) *** | 0.01 (0.07) | 0.26 (1.15) |  |
| *State 22222* | -0.2 (0.02) *** | -0.22 (0.02) *** | 0.08 (0.07) | 0.13 (1.07) |  |
| *State 23321* | -0.22 (0.02) *** | -0.25 (0.02) *** | 0.05 (0.05) | 0.25 (0.77) |  |
| *State 33323* | -0.44 (0.02) *** | -0.47 (0.02) *** | 0.01 (0.03) | 0.23 (0.49) |  |
| *State 33333* | -0.53 (0.02) *** | -0.55 (0.02) *** | - | - |  |
| *Child perspective* | 0.04 (0.01) *** | 0.03 (0.01) ** | 0.09 (0.03) ** | -0.17 (0.44) |  |
| AIC | -399.4 | -253.6 | 19.7 | 1026.9 |  |
| ****p<0.001,**p<0.01,*p<0.05* | | | | |  |
|  | | |  |  |  |
|  | |  |  |  |  |
